# Innovative aptamer approaches in glial tumor diagnostics and therapy: Progress and future directions

**DOI:** 10.1016/j.omtn.2025.102744

**Published:** 2025-10-16

**Authors:** Natalia A. Luzan, Tatiana N. Zamay, Penghui Zhang, Andrey A. Narodov, Galina S. Zamay, Anna S. Kichkailo, Maxim V. Berezovski, Olga S. Kolovskaya

**Affiliations:** 1 Federal Research Center “Krasnoyarsk Science Center” of the Siberian Branch of the Russian Academy of Sciences, Laboratory for Digital Controlled Drugs and Theranostics, Molecular Electronics Department, 660036 Krasnoyarsk, Russia; 2 Prof. V.F. Voino-Yasenetsky Krasnoyarsk State Medical University Laboratory for Biomolecular and Medical Technologies, 660022 Krasnoyarsk, Russia; 3 Zhejiang Cancer Hospital, The Key Laboratory of Zhejiang Province for Aptamers and Theranostics, Hangzhou Institute of Medicine (HIM), Chinese Academy of Sciences, Hangzhou 310022, China; 4 Department of Chemistry and Biomolecular Sciences, University of Ottawa, Ottawa, ON K1N 6N5, Canada

**Keywords:** MT: Special Issue: Innovations in Aptamer Technology, aptamer, glioma, glioblastoma, brain tumor, therapy, diagnostics, targeted delivery, theranostics

## Abstract

Glial tumors, particularly glioblastomas, remain among the most challenging cancers to diagnose and treat due to their heterogeneity, infiltrative nature, and the protective blood-brain barrier that impedes drug delivery. Aptamers—short, single-stranded nucleic acids selected for high-affinity target binding—have emerged as promising agents in neuro-oncology, offering advantages such as high specificity, low immunogenicity, and superior tissue penetration compared to conventional antibodies. This review outlines recent advancements in aptamer-based technologies for the diagnosis and treatment of glial brain tumors. We describe the use of aptamers in molecular imaging, liquid biopsy platforms, intraoperative tumor visualization, and the targeted delivery of therapeutic agents, including small molecules, small interfering RNAs (siRNAs), and immunomodulators. The integration of aptamer systems with nanotechnology and AI is accelerating the development of sensitive, non-invasive diagnostic tools and multifunctional theranostic platforms.

## Graphical abstract

**Figure:**
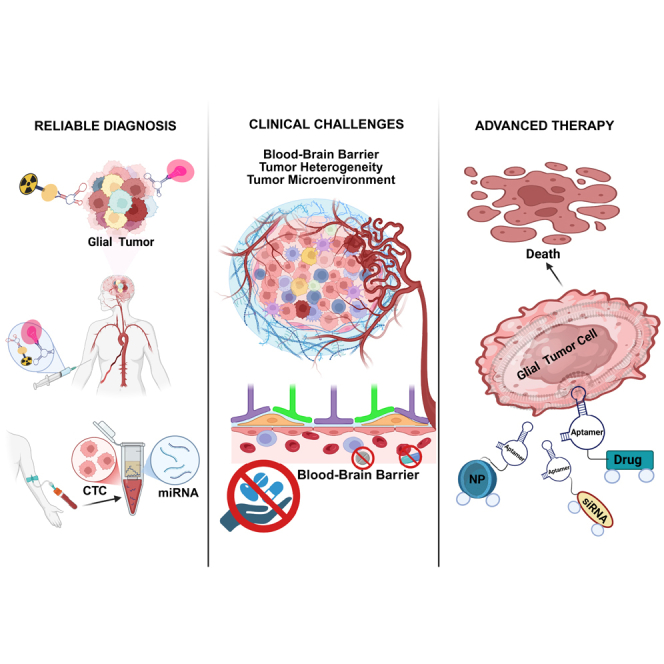

## Teaser

Aptamers are emerging as precise tools for glioma diagnostics and therapy, offering superior specificity, blood-brain barrier penetration, and versatile theranostic applications. This review highlights recent advances, clinical progress, and future perspectives of aptamer-based strategies in neuro-oncology, underscoring their potential to transform personalized brain tumor management.

## Introduction

Brain glial tumors represent a diverse group of primary central nervous system (CNS) neoplasms that arise from glial cells, which provide structural and functional support to neurons. These tumors range from slow-growing, benign forms to highly aggressive and invasive malignancies. Among them, glioblastoma (GBM) is the most common and lethal, with a median survival of roughly 14–15 months despite aggressive treatment strategies. Standard management includes maximal surgical resection followed by radiation and chemotherapy (e.g., temozolomide). However, recurrence is almost inevitable, and therapeutic options remain limited, contributing to poor long-term survival rates.^1^

One of the major challenges in the diagnosis and treatment of brain glial tumors lies in their inherent heterogeneity. The tumor microenvironment is highly complex, consisting of diverse cell populations, molecular subtypes, and a dynamic interplay between tumor cells and the surrounding normal brain tissue.^2^ Furthermore, the presence of the blood-brain barrier (BBB) restricts the penetration of many therapeutic agents, making effective drug delivery particularly challenging. Conventional diagnostic techniques, including neuroimaging using magnetic resonance imaging (MRI), computed tomography (CT), positron emission tomography (PET), and histopathological examination play a crucial role in tumor detection and classification.^3^ However, these methods have limitations in providing precise molecular characterization and predicting treatment response.

The promising therapeutic potential of aptamers was demonstrated in the phase 1/2 GLORIA trial (NCT04121455), where the PEGylated (attachment of polyethylene glycol) L-RNA aptamer NOX-A12 targeting CXCL12 improved radiotherapy effectiveness in O-6-methylguanine-DNA methyltransferase (MGMT)-unmethylated GBM. The treatment decreased tumor perfusion and produced radiographic responses in 90% of patients, with higher CXCL12 levels associated with better progression-free survival.^4^ Despite challenges in *in vivo* stability and regulatory approval, emerging chemical modifications and innovative delivery strategies are overcoming these limitations. With their high specificity, compact size, BBB penetration, and versatility in diagnostic and therapeutic applications, aptamers are positioned to improve current neuro-oncological treatments. Their unique properties enable precise molecular characterization and better treatment response prediction, paving the way for more personalized therapeutic approaches in glial tumor management.

### Classification of brain glial tumors

**Figure 1:**
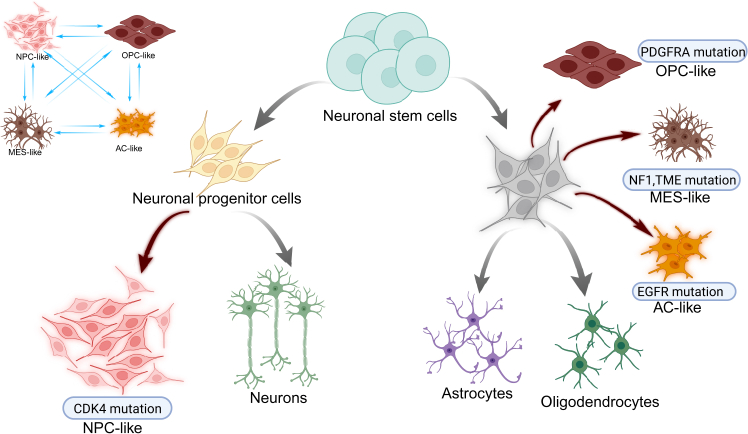
Malignant phenotypes of brain glial tumors based on the signatures of key genes Phenotypes include NPC-like, OPC-like, AC-like, and MES-like.

Brain glial tumors are classified based on histological, molecular, and genetic criteria according to the World Health Organization (WHO). The heterogeneity of high-grade glioma tumors is defined by four clinically significant subtypes based on the signatures of key genes, resulting in the identification of the four following malignant phenotypes^5^ (Figure 1):

(1) Neural stem cell-like (NPC-like): Characterized by stem cell markers (CD133/prominin-1, Oct4/POU5F1, Nanog, SOX2, NESTIN, and CD44) and neuronal differentiation pathways and predominantly localizes to the tumor core. These tumors may exhibit resistance to conventional therapies but could respond to targeted stem cell inhibitors.^6^
(2) Oligodendrocyte precursor-like (OPC-like): predominantly localize in the tumor core; show overexpression of OLIG2, platelet-derived growth factor receptor (PDGFR) alpha (PDGFRA), NG2, and SOX10, resembling oligodendrocyte progenitor cells; and are enriched at the infiltrative margin. These tumors often have a better prognosis due to higher chemosensitivity.^7^
(3) Astrocyte-like (AC-like): Exhibit astrocytic differentiation and metabolic reprogramming markers S100B, GFAP, SLC1A3, GLAST, MLC1, and AQP4. They tend to have intermediate aggressiveness.^8^
(4) Mesenchymal-like (MES-like): Associated with nuclear factor-κB pathway activation, immune infiltration, and poor prognosis. These tumors are highly aggressive and often therapy resistant.^9^

**Figure 2:**
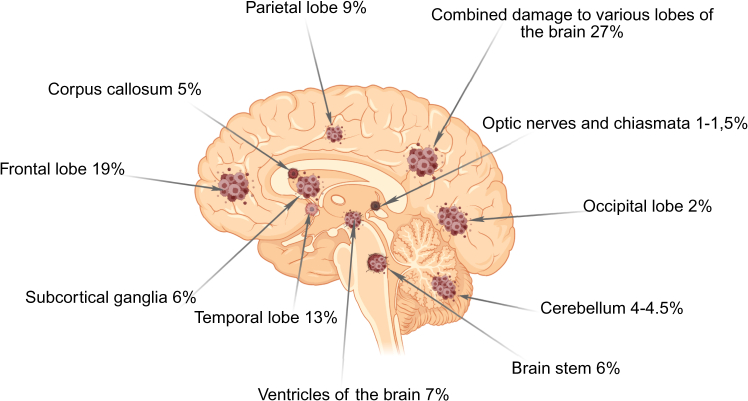
Typical localization of primary glial tumors in the brain

The molecular classification of gliomas has revealed clinically significant subtypes that correlate with tumor behavior, therapeutic response, and patient outcomes.^9^ For instance, MES-like tumors typically create immunosuppressive microenvironments, while OPC-like variants may respond to PDGFRA-targeted therapies.^10^ These molecular subtypes not only differ in their biological properties but also exhibit distinct spatial distributions within tumor tissue: NPC-like and AC-like states concentrate in the tumor core, OPC-like cells dominate the infiltrative margins, and MES-like subtypes localize to chronic hypoxic niches.^9^ Notably, undifferentiated glioma stem cells (GSCs) demonstrate remarkable plasticity, persisting across all tumor regions and adapting to diverse microenvironmental conditions.^6^ This intricate molecular and spatial heterogeneity is characteristic of gliomas—primary brain tumors arising from glial cells that are classified by their cellular origin, histological features, and increasingly, their molecular profile. The complex interplay between molecular subtypes and their anatomical distribution results in tumors that can develop throughout the CNS, with different glioma variants showing preferential localization patterns.^11^ As illustrated in Figure 2,^12^^,^^13^ these malignant foci form in specific brain regions, reflecting both the tumor’s molecular identity and its interaction with the surrounding neural tissue.

Astrocytomas, which develop from astrocytes, encompass a spectrum from low-grade (Grade I–II) to high-grade malignancies (Grade III–IV). The most indolent subtype, pilocytic astrocytoma (Grade I), is a slow-growing, often benign tumor typically found in children and young adults. In contrast, diffuse astrocytoma (Grade II) exhibits infiltrative growth and a tendency to progress to higher grades. Further progression leads to anaplastic astrocytoma (Grade III), marked by increased cellular atypia and proliferative activity.^14^ The most aggressive form, GBM (Grade IV), is characterized by rapid growth, necrosis, and a dismal prognosis. While high-grade astrocytomas are among the most lethal brain tumors, molecular profiling now plays a pivotal role in refining their classification and management.^15^

Genetic markers such as isocitrate dehydrogenase (IDH) mutations and 1p/19q co-deletion are essential in identifying tumor subtypes, determining prognosis, and guiding targeted therapies. For instance, IDH-mutant gliomas generally have a more favorable prognosis than their IDH-wild type counterparts, while 1p/19q co-deletion defines oligodendrogliomas and predicts better response to chemotherapy.^16^ Similarly, MGMT promoter methylation status is a critical biomarker in GBM, as its presence often indicates a better response to temozolomide, one of the key chemotherapies for this aggressive tumor.^17^

Oligodendrogliomas, defined by their IDH mutation and 1p/19q co-deletion status, originate from oligodendrocytes and are distinguished by their round nuclei and delicate vascular networks. The low-grade oligodendroglioma (Grade II) grows slowly but infiltrates surrounding brain tissue, while its high-grade counterpart, anaplastic oligodendroglioma (Grade III), demonstrates heightened mitotic activity and necrosis.^18^

Similarly, ependymomas arise from ependymal cells lining the ventricles and spinal cord. The benign subependymoma (Grade I) and myxopapillary ependymoma (Grade I) are slow-growing tumors typically found in the ventricles and spinal cord, respectively.^19^ In comparison, ependymoma (Grade II) exhibits moderate aggressiveness, whereas anaplastic ependymoma (Grade III) is highly malignant with a poor prognosis.^20^

**Figure 3:**
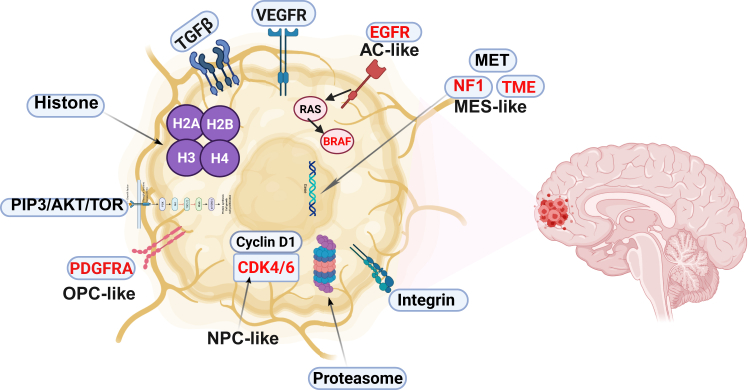
Potential biomarkers of brain glial tumors The biomarkers characterizing different cellular subtypes of glial tumors are highlighted in red—OPC-like, AC-like, MES-like, and NPC-like.

Beyond these classic glial tumors, some neoplasms display mixed or unique molecular features (Figure 3). Mixed gliomas, such as oligoastrocytomas, contain hybrid populations of astrocytes and oligodendrocytes, reflecting the complexity of glial tumorigenesis.^21^ Even more aggressive are diffuse midline gliomas, which include the devastating diffuse intrinsic pontine glioma (DIPG)—a pediatric tumor with a near-universal poor prognosis, driven by mutations like H3K27M.^22^ Advances in molecular diagnostics have revolutionized glioma classification, with markers such as IDH mutations, ∗1p/19q∗ co-deletion, MGMT promoter methylation, and ATRX loss now guiding diagnosis and treatment. These insights not only enhance prognostic accuracy but also pave the way for personalized therapeutic strategies, offering hope for improved outcomes in these challenging malignancies.

### The role of aptamers in neuro-oncology

Given the complexity and diversity of glial brain tumors, there is an increasing need for more precise and targeted diagnostic and therapeutic approaches. Aptamers, short single-stranded DNA or RNA molecules selected through systematic evolution of ligands by exponential enrichment (SELEX) for high-affinity binding to specific molecular targets, have become promising tools in neuro-oncology. They are being developed to target various molecular markers on brain tumor cells, vascular components, and microenvironmental elements. These synthetic oligonucleotides, with conformationally dynamic structures, are screened against different cell lines and primary patient-derived cells, achieving highly specific recognition through complementary surface interactions, including hydrogen bonding, electrostatic forces, and van der Waals interactions. They offer several advantages over traditional monoclonal antibodies, such as smaller size, enhanced tissue penetration, reduced immunogenicity, and more cost-effective synthesis. Aptamers are being used across multiple areas of brain tumor management, including molecular imaging, liquid biopsy-based detection, targeted drug delivery, and immunotherapy.

This review explores the current landscape of aptamer-based diagnostics and therapeutics in brain glial tumors, highlighting their mechanisms of action, advantages over conventional approaches, recent research advancements, and future clinical implications.

## Aptamers in brain glial tumor diagnostics

### Mechanisms of aptamer-based detection

Aptamers can be integrated into various diagnostic platforms to specifically recognize glial tumor cells or biomarkers (Table 1). One approach is aptamer-based histological staining, where aptamers serve as chemical antibodies to label tumor tissues. For example, an RNA aptamer named H02 selected against GBM cells (targeting integrin α5β1) was used to stain tumor biopsy samples and showed a stronger and more quantitative signal than conventional immunohistochemistry.^36^ Due to their small size, aptamers can penetrate fixed tissue more effectively than antibodies, potentially revealing antigen levels with greater sensitivity.^36^ Aptamers can also be adapted into biosensors and assays. In liquid biopsy diagnostics, aptamers have been used as capture probes in electrochemical sensors and fluorescence assays to detect tumor-derived markers such as proteins or extracellular vesicles (EVs) with high sensitivity.^37^^,^^38^^,^^39^ Notably, a number of “aptasensors” have been developed to bind circulating EVs from cancers (e.g., breast, lung), achieving their sensitive detection.^37^ In neuro-oncology, research groups are applying similar strategies to identify glioma-specific markers in cerebrospinal fluid (CSF) or blood, aiming for an earlier and less-invasive diagnosis than surgical biopsy.^39^ Another powerful application is *in vivo* imaging, where aptamers labeled with a reporter (fluorophore, radiotracer, etc.) are injected to seek out tumor cells. Once bound to their targets, these aptamer probes can be visualized by imaging modalities like MRI, fluorescence imaging, or PET, thereby highlighting tumor location and extent. For instance, the DNA aptamers Gli-55 and Gli-233 were generated to bind primary human glial tumor cells and were conjugated to imaging agents; in an animal model, they enabled fluorescence-guided visualization of xenograft tumors and could even be used for PET/CT imaging of gliomas.^3^ These mechanisms illustrate how aptamers act as highly specific detection ligands, either *ex vivo* (in tissue or biofluid samples) or *in vivo*, to improve identification of glial tumors.

**Table 1:** Potential aptamers and their targets for glioma therapy

Molecular target	Potential aptamers for the treatment of glial brain tumors	Therapeutic effect
EGFR	E07^23^^,^^24^, CL4,^25^ CL-4RNV616,^26^ anti-EGFR,^27^ U2,8,19, 31^28^	Inhibition of tumor cell proliferation, survival, angiogenesis, and invasion
VEGFR	VEGF-4-1ВВ,^29^ pegaptanib^30^	Suppression of neoplastic angiogenesis
PDGFRβ/PDGFRα	Gint4.T,^31^ PDR3^32^	Suppression of migration, invasion, expression of the transcription factor STAT3
Nucleolin	AS1411^33^^,^^34^	Suppression of angiogenesis and tumor growth
Tubulin	Gli233^3^	Reorganization of the cytoskeleton and initiation of apoptosis
CD133 (prominin 1)	CD133-A15, CD133-B19^35^	Suppression of glial stem cell viability

### Comparison with conventional diagnostic methods

Aptamer-based diagnostics offer several potential advantages over traditional methods like imaging and biopsy. Current radiographic techniques (MRI, CT, and PET) are excellent for locating brain lesions but lack molecular specificity—they cannot definitively distinguish tumor tissue from inflammation or necrosis.^3^^,^^40^^,^^41^ By contrast, an aptamer that binds a tumor-specific marker can add molecular recognition to imaging, potentially improving diagnostic accuracy. For example, an aptamer targeting an extracellular matrix protein prevalent in gliomas (tenascin-C) was radiolabeled (^99m^Tc) and showed highly specific accumulation in GBM xenografts, with tumor-to-blood signal ratios around 50:1 within hours.^42^^,^^43^ This demonstrates far greater contrast specificity than standard MRI. Likewise, while biopsy and immunohistochemistry remain the gold standard for definitive diagnosis and tumor typing, aptamer probes can augment these by detecting markers that antibodies or routine stains might miss. The H02 aptamer mentioned above could quantify integrin levels in fixed GBM specimens more dynamically than antibody staining, suggesting aptamers might reduce false-negatives or provide more accurate tumor profiling.^36^ Moreover, aptamer-based assays can be adapted to liquid biopsies, whereas conventional brain tumor diagnosis has had limited success with blood tests due to the BBB and low biomarker levels. Aptamers’ high affinity can improve the capture of rare circulating tumor cells (CTCs) or fragments. In one study, aptamers (Gli-233/Gli-55) were used to magnetically isolate CTCs from glioma patient blood samples and successfully detected tumor cells that correlate with disease.^3^ This kind of approach could complement imaging by providing a molecular diagnosis from a blood draw, something not achievable by standard methods. Overall, aptamer diagnostics strive to be less invasive (using blood or CSF instead of open biopsy), more specific (molecular targeting vs. anatomical imaging), and potentially more quantitative and rapid than some conventional assays.

### Recent advancements in aptamer-based biosensors and imaging

Cutting-edge research has produced a variety of aptamer-enabled diagnostic tools for brain tumors. One major area is fluorescence-guided surgery. To aid neurosurgeons in differentiating tumor margins, investigators have developed fluorescently labeled aptamers that bind glioma cells. For example, an aptamer called A32 that binds the EGFRvIII mutation (present in many gliomas) was conjugated to quantum dots, creating a nanoprobe that crosses the BBB and highlights EGFRvIII-positive tumor tissue with bright fluorescence.^44^ In orthotopic mouse models, this QD-aptamer probe selectively lit up EGFRvIII-expressing tumors, clearly delineating tumor boundaries during surgery, while producing no signal in normal brain or EGFRvIII-negative controls.^44^ This advancement suggests that aptamer probes could one day be used intraoperatively, similar to fluorescent dyes (like 5-ALA), but with greater tumor specificity. Another frontier is multimodal imaging. Researchers have combined aptamers with nanoparticles to enable dual imaging modalities. For instance, one group attached multiple aptamers (AS1411 against nucleolin, RGD peptide, and TTA1 against tenascin-C) onto silica-coated magnetic nanoparticles, creating a probe detectable by both optical imaging and MRI.^45^^,^^46^^,^^47^ Such probes aim to leverage aptamers’ targeting to concentrate imaging contrast agents in the tumor. On the biosensor side, aptamer-based electrochemical sensors for glioma biomarkers are being refined for higher sensitivity. Integrating AI techniques has recently been shown to significantly enhance aptamer biosensor performance. For example, AI-optimized aptamer sensors have achieved ultra-low detection limits (femtomolar range) for cancer antigens and improved specificity to >90%, far outperforming traditional antibody tests.^48^ These intelligent aptasensors can analyze complex patterns (even multiple biomarkers simultaneously) and produce rapid readouts, highlighting their potential for point-of-care diagnostics in oncology.^48^ While many of these technologies are in preclinical stages, they represent a wave of innovation that could transform how glial tumors are detected—moving toward earlier, minimally invasive, and more accurate diagnostics using aptamer tools.

#### Examples of preclinical and clinical studies

**Figure 4:**
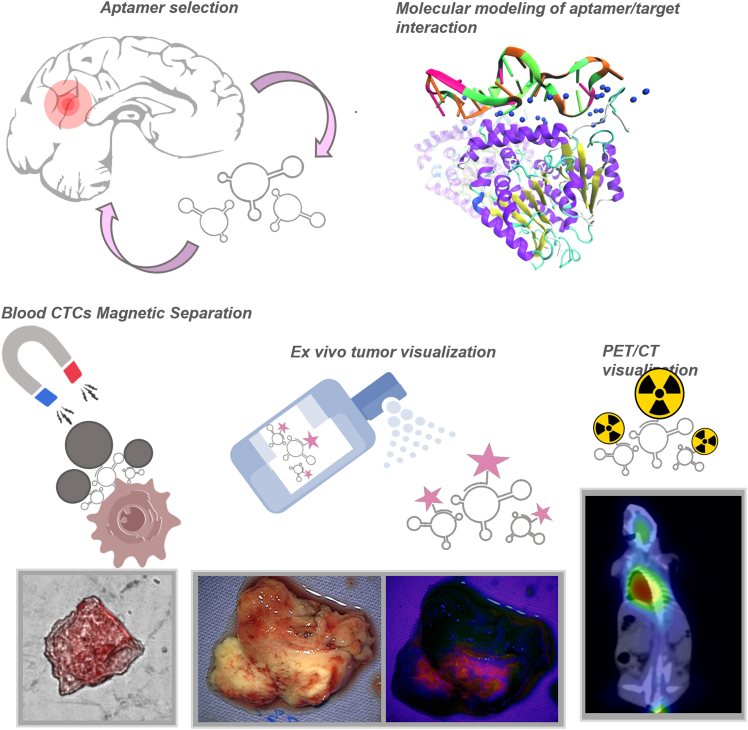
Aptamer-based strategies for glioma diagnostics (Top left) Selection of aptamers against brain tumor targets (through iterative binding to tumor cells/tissues). (Top right) Aptamer (pink/green ribbon) binding to its target protein (surface rendering) as confirmed by structural modeling. (Bottom left) Aptamers immobilized on magnetic beads can capture circulating tumor cells (CTCs) from blood for analysis. (Bottom middle) *Ex vivo* application of fluorescent aptamers to resected tumor tissue allows visualization of tumor margins. (Bottom-right) *In vivo* PET/CT imaging of a mouse with a glioma (green hotspot) after injection of a radiolabeled aptamer, demonstrating specific tumor targeting. The figure was adopted from Kichkailo et al.^3^

Numerous proof-of-concept studies underscore the diagnostic potential of aptamers in neuro-oncology. In a 2023 report by Kichkailo et al., two DNA aptamers (Gli-233 and Gli-55) were selected through cell-SELEX using patient-derived glioma cells and shown to bind specifically to high-grade glioma tissues.^3^ These aptamers successfully detected CTCs in blood samples from glioma models and, when labeled, enabled both fluorescence imaging and PET/CT of glioma xenografts^3^ (Figure 4). Importantly, they exhibited no toxicity in mice,^3^ supporting their safety for potential clinical translation. Another example is the TTA1 aptamer (RNA aptamer to tenascin-C): in a classic study, TTA1 was radiolabeled and injected into mice bearing GBM xenografts, where it rapidly homed to tumors within 10 min and showed sustained tumor retention over 18 h.^37^^,^^42^ This resulted in high-contrast single-photon emission CT imaging of the gliomas, validating the concept of aptamer-guided nuclear imaging. On the clinical front, aptamer-based diagnostics are still emerging. Platforms like the SOMAscan (an aptamer-based proteomic assay) can profile thousands of proteins in patient biofluids^38^^,^^39^; while not yet applied specifically to brain tumors, this technology could potentially identify glioma biomarkers in blood or CSF with unprecedented breadth and precision. To date, no aptamer diagnostic has been formally approved for brain tumors, but early-phase clinical studies are anticipated. The encouraging preclinical data—ranging from aptamer immunohistochemistry achieving double the sensitivity of antibodies^37^ to aptamer probes enabling non-invasive imaging of gliomas—lay the groundwork for translating aptamer diagnostics into clinical trials. These advances suggest that in the near future, aptamers might complement MRI and biopsy by providing new molecular diagnostic avenues for glial tumors.

## Aptamer-based therapeutic strategies

Aptamers have emerged as powerful delivery platforms in neuro-oncology, enabling targeted transport of diverse therapeutic agents. These chimeric constructions include aptamer-small interfering RNA (siRNA) conjugates: (1) the PDGFRβ-targeting Gint4.T aptamer delivers *STAT3 siRNA* to GBM cells, achieving significant gene knockdown and tumor growth inhibition,^49^ and (2) AS1411 and a CpG ODN targeting nucleolin and TLR9 deliver siRNA to osteopontin.^50^ Another approach employs aptamer-drug conjugates (ApDCs), such as the GD2-binding DB99 aptamer, which co-deliver doxorubicin and MYCN siRNA in neuroblastoma, showing synergistic efficacy.^51^ The bifunctional TEPP aptamer (TfR/EpCAM targeting) delivers *doxorubicin* across the BBB to brain metastases.^52^ Aptamers can be utilized in conjunction with nanoparticles. RA16-PEGylated nanoparticles encapsulating epirubicin enhance circulation time and tumor accumulation in NSCLC.^53^ Finally, aptamers can possess intrinsic therapeutic efficacy. For example, the anti-EGFR aptamer CL4 directly inhibits tyrosine kinase signaling in GBM.^54^ These platforms address key neuro-oncology challenges: overcoming poor biodistribution, reducing off-target effects, and enabling CNS access through the BBB.

### Mechanisms of aptamer-mediated therapy

Beyond diagnostics, aptamers are being harnessed as therapeutic agents or delivery vehicles to combat glioma tumors. One mechanism is the direct inhibition of tumor growth signals. Aptamers can function as antagonists by binding and blocking receptors or ligands vital to tumor proliferation. For example, an RNA aptamer termed CL4 that binds the EGFR receptor was shown to also recognize the mutant EGFRvIII in GBM cells; upon binding, CL4 inhibited EGFRvIII autophosphorylation and downstream signaling, which led to reduced migration and proliferation of GBM cells.^37^ Similarly, the DNA aptamer Gint4.T targets PDGFRβ—a key growth driver in mesenchymal GBM—and not only binds the receptor but also interferes with its activity, effectively acting as an anti-cancer agent on its own.^37^ A second powerful approach is targeted drug delivery using aptamers as homing devices. Aptamers can be conjugated to a therapeutic payload (small molecule drug, toxin, siRNA, radionuclide, etc.) to selectively deliver it to tumor cells, minimizing systemic toxicity. In a striking demonstration, researchers designed an “aptamer-siRNA chimera” by attaching the Gint4.T aptamer to a siRNA against the STAT3 gene.^37^ The Gint4.T portion guided the complex into PDGFRβ-expressing GBM cells, bringing the STAT3 siRNA inside those cells. This chimera achieved efficient STAT3 gene silencing *in vitro* and significantly suppressed tumor growth and angiogenesis *in vivo* in a GBM xenograft model.^37^ Such chimeric molecules use aptamers as a Trojan horse to smuggle therapeutic RNA into cancer cells. Likewise, aptamers have been used to ferry chemotherapeutic drugs: one strategy is to chemically link a drug (like doxorubicin) to an aptamer or to intercalate it into a DNA aptamer’s structure so that the drug is released primarily at the tumor site when the aptamer binds its target. Another novel example is aptamer-guided gene therapy: scientists inserted the AS1411 DNA aptamer (which targets nucleolin on cancer cells) into a plasmid encoding a potent toxin protein and delivered this construct using nanoparticles to glioma cells. The aptamer moiety directed the toxin gene specifically into nucleolin-expressing tumor cells, resulting in selective intracellular production of the toxin and cell death.^37^ Non-tumor cells lacking surface nucleolin were spared, showing the precision of aptamer targeting. Finally, aptamers are being explored in immunotherapy for gliomas. Immune checkpoint blockade with monoclonal antibodies has had limited success in most gliomas, partly due to the immunosuppressive tumor microenvironment. Aptamers can be engineered to bind immune checkpoints or modulatory receptors in a similar way. A recent study used an RNA aptamer against TIM-3 (an inhibitory receptor on T cells and other immune cells) to treat diffuse midline glioma, a lethal pediatric brain tumor. The TIM-3 aptamer by itself enhanced T cell infiltration in the tumor and showed a trend of prolonged survival in mice; when combined with radiation therapy, it significantly increased median survival and produced some long-term survivors, an effect lost if T cells were depleted.^37^ This indicates aptamers can function as immunotherapeutic agents, either alone or as adjuvants to standard treatments like radiotherapy. In summary, aptamer-mediated therapies act through a variety of mechanisms—from directly blocking oncogenic pathways, to smart delivery of drugs/genes, to modulating the immune response—all aiming to specifically target and kill tumor cells while sparing healthy brain tissue.

### Comparison with traditional therapeutic modalities

Aptamer-based therapeutics offer a unique blend of advantages compared to conventional treatments. Unlike small-molecule drugs, which often lack specificity and can affect both tumor and normal cells, aptamers can be selected for extremely high specificity to a tumor-associated target, reducing off-target effects.^55^ This specificity is on par with or better than monoclonal antibodies, yet aptamers are much smaller (∼10× smaller than IgG antibodies).^50^^,^^56^^,^^57^^,^^58^^,^^59^^,^^60^^,^^61^^,^^62^^,^^63^ The small size confers better tumor penetration—crucial in an infiltrative glioma—and, importantly for neuro-oncology, potentially allows aptamers to cross the BBB where large antibody molecules cannot.^42^^,^^55^^,^^61^^,^^64^^,^^65^^,^^66^^,^^67^^,^^68^ Indeed, modeling studies predict that an aptamer may achieve 100-fold higher brain uptake than an antibody of the same dose.^37^^,^^61^ Another comparison is immunogenicity: protein therapeutics (antibodies, some peptides) can trigger immune reactions or lose efficacy due to neutralizing antibodies. Aptamers, being nucleic acids, typically do not elicit an immune response in humans,^63^^,^^69^^,^^70^^,^^71^^,^^72^ which is a significant advantage for repeated dosing or long-term treatments. They also do not accumulate in the body (since nucleic acids are eventually metabolized), potentially reducing long-term toxicity. In contrast, traditional cytotoxic drugs cause systemic side effects (e.g., temozolomide, which causes bone marrow suppression) because they cannot distinguish tumor cells from healthy proliferating cells. ApDCs, by delivering chemotherapeutics straight to tumor cells, aim to maximize drug concentration at the tumor while minimizing exposure elsewhere.^55^^,^^65^^,^^73^ This targeted approach is akin to antibody-drug conjugates used in oncology, but aptamers are synthetic and easier to produce and modify chemically. Another area is gene therapy: viral vectors have been explored to deliver genes to gliomas, but targeting is a hurdle and off-target transduction can be dangerous. Aptamer-siRNA chimeras or aptamer-decorated nanoparticles offer a non-viral, targeted gene delivery platform, avoiding some safety issues of viruses while improving localization to tumor cells.^49^ Even in the realm of immunotherapy, aptamers have some perks. For example, an aptamer antagonist to a checkpoint can be synthesized quickly, and its activity can be turned off by administering a complementary “antidote” oligonucleotide that binds the aptamer, providing a way to stop therapy in case of adverse effects.^74^ Monoclonal antibodies cannot be so easily neutralized once given. Of course, traditional therapies have their own strengths—small molecules often have oral availability (whereas aptamers must be injected) and antibodies have long half-lives in circulation (whereas aptamers may clear rapidly if not modified). However, in the challenging landscape of brain tumor treatment, the precision and versatility of aptamer-based strategies offer a compelling complementary approach. They strive to overcome the limitations of existing modalities by ensuring that therapeutic action is concentrated at the tumor site, potentially increasing efficacy and reducing toxicity in a way that traditional drugs or biologics have struggled to achieve in neuro-oncology.^42^^,^^55^^,^^64^^,^^65^^,^^66^^,^^67^^,^^68^^,^^73^^,^^75^^,^^76^^,^^77^^,^^78^

### Current progress in preclinical and clinical research

**Figure 5:**
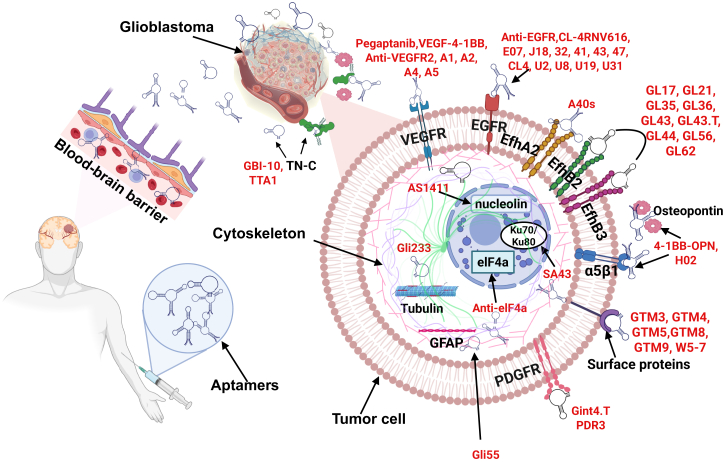
Aptamers and their molecular cellular targets for diagnostics and therapy of human brain glial tumors

Aptamer therapeutics for glial tumors are advancing through preclinical studies and beginning to enter clinical evaluation. On the preclinical side, a rich pipeline of aptamers targeting various GBM-associated molecules has shown promising results in cell and animal models. Various molecular targets of GBM and some of the aptamers obtained against them are presented in Figure 5. Besides the earlier examples (EGFRvIII, PDGFRβ, etc.), aptamers have been developed against other glioma markers like Axl (a receptor tyrosine kinase), IL-6 (a cytokine in the tumor microenvironment), and extracellular matrix components, demonstrating the adaptability of aptamers to different targets.^58^ Combination strategies are also being tested: given the network redundancy in GBM, using two aptamers to concurrently block, say, EGFRvIII and PDGFRβ has been suggested to prevent compensatory pathway activating.^37^ One study showed that treating EGFRvIII-expressing GBM cells with the CL4 aptamer (anti-EGFR) led to the upregulation of PDGFRβ as a resistance mechanism, implying that a dual-aptamer therapy (EGFR + PDGFRβ) could be more effective.^37^ In live models, ApDCs are yielding encouraging outcomes. For instance, anti-nucleolin aptamer AS1411 has been used to deliver nanoparticles loaded with chemotherapeutics across the BBB, resulting in inhibited tumor growth in orthotopic glioma mice compared to non-targeted nanoparticles.^43^^,^^45^^,^^46^ Importantly, some aptamers have progressed to clinical trials in oncology. AS1411 was one of the first cancer aptamers tested in humans: in phase 1 and 2 trials involving patients with advanced solid tumors, AS1411 showed signs of anti-cancer activity with minimal toxicity.^79^^,^^80^^,^^81^ Although those trials were not brain specific, they demonstrated the safety of aptamer therapy in humans. In neuro-oncology specifically, an L-aptamer (Spiegelmer) named NOX-A12 (olaptesed pegol) is currently in a phase 1/2 trial (the GLORIA trial) for patients with newly diagnosed GBM. NOX-A12 binds and neutralizes CXCL12 (SDF-1), a chemokine that attracts immunosuppressive cells and promotes tumor invasion. The trial combines NOX-A12 with standard radiotherapy in GBM patients with unmethylated MGMT (who derive little benefit from temozolomide).^4^ Interim results have shown the aptamer is well tolerated and indicated favorable safety and preliminary efficacy, such as signs of prolonged survival, compared to historical controls with radiotherapy alone.^4^ Another aptamer, CpG-ODN (although not a targeting aptamer but an immunostimulatory DNA sequence), has been injected into gliomas in trials to activate immune responses, underscoring the clinical interest in nucleic acid therapeutics for brain tumors. Moving forward, the TIM-3 aptamer for pediatric diffuse gliomas is poised for a first-in-human trial in combination with radiation, after its striking preclinical success.^74^ As of now, over 30 clinical trials of aptamers for various indications have been launched, including about eight in cancer.^37^ These include aptamers being used as standalone treatments, in combination with chemo/radiotherapy, or as precision delivery agents. While we await results in the brain tumor domain, the growing body of translational research is laying crucial groundwork. The next few years may see aptamer-based therapies entering clinical practice for aggressive gliomas, especially if they continue to show the ability to hit targets that traditional drugs cannot reach and to do so safely. Some aptamers selected to treat or diagnose human brain glioma cells are presented in Table 2.

**Table 2:** Aptamers for the diagnosis and therapy of human gliomas

Aptamer	Target	Modification	Using	References
**Molecular target: tenascin-C**				

GBI-10	C6 glioma cells and U251 cell line	^8^F; ^64^Cu; 2′-deoxyinosine and D-/L-isonucleoside; quantum dots; adenovirus; magnetic nanoparticles	Identification of glial cells; PET/CT; determination of the strength of interaction between aptamers and a molecular target; *in vitro* identification for human glioma cells of the U251 line; gene therapy; MRI diagnostics of tumors	Jacobson et al.^82^; Fan et al.^83^
TTA1	Human GBM cells line U251; tenascin-C protein	Fluorescent labels; phosphorus-32; gold nanorods	*In vivo* tumor imaging using plasmon resonance, fluorescent (red) and radioisotope (99mTc) labeling	Hicke et al.^73^

**Molecular target: receptors EphB3, EphB2, EphA2**				

GL17, 21, 35, 36, 43, 43.T, 44, 56, 62	EphB3 и EphB2; U87-MG	Shortening	Inhibition of ERK 1/2 and cyclin D1; blockade of GBM cell proliferation and migration	Amero et al.^84^
A40s	Glial stem cells	–	Tumor growth suppression; migration suppression	Affinito et al.^85^

**Molecular target - receptors – EGFR/EGFRvIII**				

E07	Proteins EGFR and EGFRvIII	FAM	Receptor inhibition; suppression of EGFR autophosphorylation and proliferation in culture; tumor cell sorting and detection; identification of brain tumor cells *in vitro* using fluorescence microscopy; dynamic morphology; identification of CTCs	Hasan et al.^23^; Li et al.^24^
CL4	A549 cells	–	EGFR inhibitor; stimulation of glial cell apoptosis; suppression of proliferation	Camorani et al.^25^
J18	Protein EGFR	–	Isolation of CTCs; targeted delivery of gold nanoparticles	Wan et al.^86^
CL-4RNV616	U87MG cells	–	*In vitro* tumor growth suppression; induction of apoptosis	Wang et al.^26^
32, 41, 43, 47	U87MG-EGFRvIII cells	Fluorescent label; 188Re; siRNA; quantum dots	Tumor cell imaging; *in vivo* glioblastoma imaging; targeted delivery of siRNA by endocytosis; suppression of c-Met target gene expression and modulation of U87-EGFRvIII cell apoptosis and proliferation; intraoperative imaging	Tang et al.^44^; Wu et al.^87^
Anti-EGFR	GBM cell lines	–	Dynamic morphology	Mahmood et al.^27^
U2,8,19, 31	U87 MG	188Re; gold nanoparticles	Tumor imaging in mice using single-photon emission CT; suppression of U87 MG cell proliferation, migration, invasion, and signaling; inhibition of tumor growth *in vitro* and prolongation of survival *in vivo*	Peng et al.^28^

**Molecular target: PDGFRβ/PDGFRα**				

Gint4.T	Human glioblastoma cell line, U87 MG; glial stem cells	MicroRNA; tFNA-paclitaxel; PI3K-mTOR inhibitor	Suppression of signal transduction, migration, tumor growth, induction of differentiation *in vivo*; targeted delivery of microRNA to GBM cells; inhibition of STAT3 *in vivo*; induction of apoptosis	Shi et al.^88^
PDR3	PDGFRα	MicroRNA	Inhibition of STAT3 transcription factor expression	Yoon et al.^32^

**Molecular target: VEGFR**				

Anti-VEGFR2	Glial stem cells	Magnetic nanoparticles	Determination of the oncogenic potential of tumor stem cells using MRI	Shi et al.^88^
A1, A2, A3, A4, A5	Glial stem cells; human GBM cell line U87 MG; CD133+ cells	Magnetic nanoparticles; Cy5 fluorescent label	*In vivo* imaging of U87 MG cells using MRI and fluorescence microscopy	Kim et al.^89^
VEGF-4-1ВВ	Glial tumor	–	Antitumor immunotherapy	McNamara et al.^29^
Pegaptanib	Protein VEGF	PEG	*In vivo* reduction of tumor blood vessel density	Verhoeff et al.^30^

**Molecular target: nucleolin**				

AS1411	C6 glioma models; human GBM cell line U87 MG	Magnetic nanoparticles; Cy3 fluorescent labels; TGN peptide; docetaxel; gold nanoparticles; quantum dots	*In vivo* imaging of C6 glioma using fluorescence and MRI; targeted delivery of paclitaxel; increased survival of rats with C6 gliomas; transport across the BBB and targeted delivery of a chemotherapeutic agent; active transport into GBM tissue *in vivo*; proapoptotic effect; quantum dot nanoprobe for molecular imaging of U87 MG cells	Luo et al.^33^; Alibolandi et al.^34^

**Molecular target: endothelial protein pigpen**				

Aptamer III.1	Transformed rat endothelial cells	–	Identification of tumor microvessels to characterize the transition from quiescence to angiogenesis	Blank et al.^90^

**Molecular target: DNA repair proteins Ku 70 and Ku 80**				

SA43	Human GBM cell line U87MG	–	Differentiation of GBM cells and non-malignant cells	Aptekar et al.^91^

**Molecular target: midkine**				

Anti- midkine	Helicase	–	Midkine inhibitor; suppression of conformational changes in eIF4A helicase domains	Oguro et al.^92^

**Molecular target: integrin α5β1**				

H02	U87MG α5+	Fluorescent labels Cy5, Alexa 564	*In vitro* and *ex vivo* imaging using confocal microscopy, SPR	Fechter et al.^36^

**Molecular target: tubulin**				

Gli-233	Postoperative human glial tumor tissues	Fluorescent labels Cy5, Alexa 564	CTC detection; aptahistochemistry; GBM identification PET/CT *in vivo*	Kichkailo et al.^3^

**Molecular target: transferrin receptor**				

Aptamer TfR	Human GBM cell line U87 MG	RNV541	Suppression of expression miR-21	Larcher et al.^93^
GS24	Glial tumor	tFNA-TMZ	Tumor growth suppression *in vitro*, *in vivo*	Fu et al.^94^

**Molecular target: CD133**				

CD133-A15, CD133-B19	HEK293T-CD133; glial stem cells	–	Glial stem cells	Shigdar et al.^35^
A40s	Glial brain tumors	miRNA; anti-miRNA; Cy5; Alexa 488	Visualization of GBM cells	Affinito et al.^85^

**Bivalent aptamer. Molecular targets are osteopontin and integrin**				

4-1BB-OPN	Glial brain tumors	–	*In vivo* immunostimulation; increased survival	Wei et al.^95^

**Molecular target: cell surface proteins or associated molecules**				

GTM3, 4, 5, 8, 9	GBM cell line A172	Fluorescent labels	Tumor imaging *in vitro*	Bayrac et al.^96^
W5-7	Glial stem cells	Molecular probe	Detection and isolation of GBM cells	Wu et al.^97^

**Molecular target: glial fibrillary acidic protein**				

Gli55	Postoperative human glial tumor tissues	Fluorescent labels Cy5, Alexa 564	CTC detection; apthastochemistry; GBM identification PET/CT *in vivo*	Kichkailo et al.^3^

**Aptamers that penetrate into cells with an unknown target**				

WQY-9, 9-B	Gliosarcoma cell line K308	–	Internalization into target cells	Wu et al.^97^

**Aptamers with unknown target**				

WYZ-37, 41, 50, 41a, 50a	Target cells T98G, A172	Fluorescent labels Cy5 and FITC	Target cell recognition T98G	Wu et al.^98^
GMT8	Human GBM cell line U87 MG	Nanoparticles from polyethylene glycol-poly-ε-caprolactone	Targeted delivery of docetaxel; apoptosis of U87 MG cells *in vitro* and *in vivo;* suppression of tumor growth	Gao et al.^99^
GMT-3	Cell line GBM A-172	Doxorubicin	Targeted delivery of doxorubicin	Bayraç et al.^100^
GBM128и 131	Cell line U118 MG	Fluorescent labels	GBM imaging	Kang et al.^101^
S6-1b	SHG44, GBM	Fluorescent labels	Diagnostic and therapeutic potential to specifically deliver imaging or therapeutic agents to malignant gliomas	Lin et al.^102^
Gli233, Gli55	Primary culture of GBM	Fluorescent labels	Fluorescence imaging of tumors *ex vivo*, CTC search.	Zamay et al.^103^

CTC, circulating tumor cell; FITC, fluorescein isothiocyanate; SPR, surface plasmon resonance.

## Advantages and challenges

Aptamer technology offers many advantages for brain tumor diagnostics and therapy, but it also encounters significant challenges that need to be addressed for clinical success. Below, we summarize the main benefits of aptamers over traditional approaches, along with their limitations and strategies to overcome them.

### Advantages of aptamers in neuro-oncology

• High specificity and affinity

Aptamers can be selected to bind virtually any molecular target (proteins, cell markers, etc.) with nanomolar or even picomolar affinity, comparable to or better than antibodies.^37^^,^^64^^,^^65^^,^^73^^,^^104^^,^^105^^,^^106^^,^^107^ This allows highly specific recognition of tumor-associated markers, translating to precise diagnostics or targeting of therapy.

• Small size and tissue penetration

At ∼10–30 kDa, aptamers are an order of magnitude smaller than monoclonal antibodies (∼150 kDa). Their compact size facilitates deeper penetration into tumor tissue and better traversal of the BBB.^50^^,^^56^^,^^57^^,^^58^^,^^59^^,^^60^^,^^61^^,^^62^^,^^63^^,^^64^^,^^65^^,^^73^^,^^104^^,^^105^^,^^106^^,^^107^ This is crucial in treating infiltrative glial tumors and reaching tumor cells that reside behind an intact BBB.

• Low immunogenicity

As oligonucleotides, aptamers are generally non-immunogenic and do not trigger significant immune responses.^63^^,^^64^^,^^65^^,^^69^^,^^70^^,^^71^^,^^72^^,^^73^ They lack the protein structure that would typically be seen as foreign by the immune system, which means repeat administrations are less likely to cause allergic reactions or neutralizing antibodies.

• Chemical stability and modifiability

Aptamers can be chemically synthesized with high purity and stability. They are stable over a range of temperatures and storage conditions, with a long shelf life, unlike fragile antibodies. Moreover, they can be easily modified (e.g., adding fluorescent dyes, radiolabels, drug conjugates, or backbone modifications) without losing function, giving flexibility in designing diagnostics or therapeutics.^42^^,^^55^^,^^65^^,^^66^^,^^67^^,^^68^^,^^73^

• Reproducible and cost-effective production

Aptamer production does not rely on living cell cultures—it is a cell-free chemical process, which ensures batch-to-batch consistency and scalability.^64^^,^^65^^,^^73^^,^^104^^,^^105^^,^^106^^,^^107^ This can drive down costs; aptamers can often be produced faster and cheaper than monoclonal antibodies, and manufacturing is easily standardized (akin to synthetic drugs).

• Versatility of applications

The same aptamer can be adapted for multiple purposes—for instance, an aptamer that binds a GBM cell surface marker could be used for imaging, for capturing cells in a biosensor, or for delivering a therapeutic payload. This multi-functionality means aptamers can serve as a single platform that unifies diagnosis and therapy (so-called theranostics), tailored by simple conjugation of different agents.

### Challenges and limitations in clinical translation

• Nuclease degradation and short half-life

Unmodified RNA or DNA aptamers are susceptible to degradation by nucleases in blood and tissues, leading to a short circulating half-life and reduced efficacy.^37^ They are also small enough to be rapidly filtered out by the kidneys. This means aptamers, in their native form, may not persist long enough *in vivo* to reach their tumor targets in sufficient concentrations.

• Need for chemical modifications

To combat instability, aptamers often require chemical modifications, such as 2′-fluorine or 2′-O-methyl substitutions on the ribose, phosphorothioate backbones, or capping the ends (e.g., with an inverted nucleotide).^37^ Additionally, PEGylation or other macromolecules can increase an aptamer’s molecular weight to reduce renal clearance.^37^ While these modifications greatly improve nuclease resistance and half-life, they add complexity to manufacturing and regulatory approval.

• Potential immunogenicity of modifications

Aptamers themselves are non-immunogenic, but certain modifications can introduce issues. For example, PEGylated aptamers have, in some cases, triggered anti-PEG antibody responses that reduce the aptamer’s efficacy.^37^ Care must be taken in designing aptamer conjugates to avoid carriers or motifs that the immune system might recognize.

• Off-target binding and toxicity

If an aptamer’s target is not tumor specific, it may bind to normal tissues expressing the antigen and cause off-target effects. High-affinity aptamers could conceivably interfere with normal physiological processes if their targets are present in healthy cells.^63^^,^^69^^,^^70^^,^^71^^,^^72^^,^^108^ Rigorous negative selection during SELEX and thorough preclinical specificity testing are necessary to ensure that aptamers do not have problematic cross-reactivities.

• Delivery and distribution challenges

While aptamers can penetrate tissues better than antibodies, delivering them to all tumor cells (especially in a heterogeneous, diffuse glioma) is still a challenge. Some regions of the tumor may have an intact BBB that even aptamers struggle to cross. Additionally, aptamers may be distributed into compartments like the CSF and cleared before homing to the tumor.^37^ Novel delivery systems (e.g., aptamer-loaded nanoparticles or convection-enhanced delivery directly into the brain) are being explored to address this.

• Regulatory and development hurdles

The field of aptamers is relatively young, and currently, only two aptamer-based drugs have been approved: pegaptanib for macular degeneration and avacincaptad pegol for the treatment of geographic atrophy. Clinical trials of aptamers in cancer have had mixed results—some showing safety but limited efficacy.^37^^,^^79^^,^^81^ Regulatory agencies will require robust evidence of durable benefits in patients. This means aptamer therapeutics must clear the same high bar of clinical trial success as any drug, which can be time consuming and costly. The path to commercialization also depends on pharmaceutical investment; historically, some aptamer-focused companies struggled, which slowed development. However, with improving technology and successful examples accumulating, there is renewed momentum to bring aptamer innovations to market.

To overcome many of these challenges, researchers are devising solutions such as spiegelmers (L-enantiomer aptamers resistant to nucleases), smart delivery conjugates (e.g., aptamer-nanoparticle hybrids that prolong circulation time), and more stringent SELEX strategies to improve specificity. Encouragingly, preclinical and early clinical studies have demonstrated that with appropriate modifications, aptamers can be made sufficiently stable and effective *in vivo*.^37^ The continued refinement of aptamer design and delivery will be critical in translating their laboratory promise into clinical reality.

## Future perspectives and clinical implications

Aptamer-based diagnostics and therapeutics for brain tumors are on the cusp of important developments, and several emerging trends suggest their impact will grow in the coming years. One major trend is the push toward personalized and precision medicine using aptamers. Because aptamers can be selected against virtually any target, it is conceivable to generate patient-specific aptamers that bind to an individual’s tumor. Advanced SELEX methods are being explored where aptamer selection is performed *in vivo* or on fresh patient-derived xenografts (PDX) to yield binders that recognize the actual patient tumor environment.^37^^,^^109^^,^^110^^,^^111^ Such in vivo-SELEX could produce aptamers that inherently navigate the BBB and seek out tumor cells in the brain, a level of optimization not possible with standard drug screens. In the future, we may see “aptamer libraries” tailored to subtypes of gliomas—e.g., a set of aptamers specific for IDH-wild-type GBM vs. IDH-mutant tumors—enabling a form of precision targeting based on a tumor’s molecular profile.^109^^,^^110^^,^^111^ These aptamers could be conjugated to a variety of therapeutic payloads or imaging agents, essentially creating a customizable toolkit for each patient’s tumor characteristics. This vision aligns with the broader trend of personalized oncology, wherein treatments are adapted to the unique genetic and antigenic makeup of each cancer.

Another promising avenue is the integration of aptamer technology with AI and big data. AI-driven approaches can greatly assist in the design and application of aptamers. Machine learning algorithms are now being used to analyze large datasets from SELEX experiments to identify optimal aptamer sequences and predict their binding structures, accelerating the discovery of high-affinity aptamers for difficult targets (like mutant proteins or glycosylation-specific epitopes). On the diagnostic side, AI has been combined with aptamer-based sensors to enhance performance. As noted, AI-optimized aptasensors have demonstrated markedly improved sensitivity and reduced error rates in detecting cancer biomarkers.^48^ In the context of brain tumors, this could translate to aptamer biosensors capable of picking up trace amounts of a glioma-specific marker in blood or CSF with high reliability, something that might have been unattainable before. AI can also help interpret multiplexed aptamer assays (for instance, SOMAscan data, which yields thousands of protein measurements) to find patterns diagnostic of tumor presence or recurrence.^37^^,^^38^ In imaging, AI algorithms could be used to analyze aptamer-based imaging results intraoperatively, instantly differentiating tumor from normal tissue fluorescence to guide surgeons. Thus, the fusion of aptamers with AI and computational tools stands to refine both the selection process and the end use of aptamers in clinical diagnosis.

We also anticipate a broader expansion of aptamer theranostics—single agents or paired agents that can both image and treat tumors. For example, an aptamer that targets a GBM cell surface protein could be labeled with a PET isotope for imaging to identify the extent of disease and the same aptamer could be attached to a radioisotope or toxin for therapy. This kind of dual use could streamline the pipeline from diagnosis to treatment, making personalized medicine more seamless. Additionally, aptamers might be integrated with other innovative therapies. One could envision aptamer-targeted delivery of emerging treatments like CRISPR-Cas9 gene editors or novel immunostimulatory RNAs to brain tumors. By ensuring these powerful tools only affect cancer cells, aptamers could help mitigate risks and increase efficacy. From a clinical and regulatory standpoint, the coming years will test the viability of aptamer-based drugs and diagnostics in practice.

As multiple aptamer candidates progress through trials, positive outcomes will be crucial to gain regulatory approvals. The GLORIA trial of the NOX-A12 aptamer in GBM, for instance, is a significant litmus test. If it demonstrates improved survival or patient outcomes when final results are analyzed, it could pave the way for the first approved aptamer therapy in neuro-oncology.^4^ Similarly, aptamer diagnostic tools will need to show that they meaningfully add accuracy or clinical utility beyond current standards (e.g., detecting tumor recurrence earlier than MRI or stratifying patients by likely responders to a therapy). Regulatory agencies will scrutinize factors such as manufacturing consistency, safety (especially of any chemical modifications), and demonstration of benefit in well-designed studies. The good news is that the field has matured considerably: the past failures and challenges have informed better designs, and there is now a track record of safe aptamer use in humans.^37^^,^^81^ Commercialization efforts are also ramping up—companies are developing platforms for rapid aptamer generation and some are focusing on ApDCs as a new class of targeted therapeutics. Aptamers could also find a niche in combination with existing treatments. For instance, using an aptamer to deliver a radiosensitizer into GBM cells could enhance the effect of radiotherapy (much like the TIM-3 aptamer did by modulating the immune environment for radiation).^74^ The outlook is that aptamers will not replace conventional treatments outright but rather augment and improve the precision of brain tumor management. In a decade’s time, a glioma patient’s care might involve an aptamer-based PET scan to map the tumor, an aptamer-linked drug to target residual tumor cells after surgery, and perhaps an aptamer immunotherapy to stimulate the immune system against the cancer. This level of targeted approach could significantly shift the paradigm toward better outcomes.

Finally, aptamer research is extending into previously uncharted territories like pediatric brain tumors and rare glial neoplasms. The specificity of aptamers could be especially beneficial in children, where sparing normal brain tissue is paramount. Targets like the H3K27M mutation in diffuse midline gliomas (DIPG) are being considered for aptamer development,^109^^,^^110^^,^^111^ since traditional antibody therapies cannot easily distinguish this mutant histone. Success in such difficult diseases would underscore aptamers’ value. In conclusion, the future of aptamers in neuro-oncology is bright, built on a foundation of innovation at the intersection of molecular biology, engineering, and computational science. Their continued evolution promises to bring neuro-oncologists closer to the long-sought goals of early detection, precise targeting, and effective eradication of brain tumors with minimal collateral damage.

## Conclusion

Aptamers have rapidly emerged from a novel laboratory concept to a versatile tool with tangible applications in the fight against brain glial tumors. In diagnostics, aptamer-based strategies are addressing long-standing challenges by enabling more specific tumor detection—whether through improved histological staining, minimally invasive liquid biopsies, or high-contrast imaging of gliomas. In therapeutics, aptamers offer a means to surmount the BBB and tumor heterogeneity, acting as targeted delivery vehicles and potent antagonists of cancer pathways. Through multiple examples, we have seen that aptamers can achieve targeted binding and therapeutic effects that rival or exceed traditional antibodies and small molecules in preclinical models.^42^^,^^49^ Key advantages such as low immunogenicity, ease of synthesis, and flexible modifiability give aptamers an edge in designing personalized treatment regimens, while challenges like nuclease stability and *in vivo* delivery are being actively mitigated with chemical innovations and smart formulations. Current research trends point toward the integration of aptamers into a broader personalized medicine framework, where they may be custom selected for a patient’s tumor profile and combined with AI-driven diagnostic algorithms for optimal care. Although predominantly at the experimental and early clinical stage now, aptamer technology is steadily advancing on the translational path. The next generation of clinical trials, including those for aggressive entities like GBM and diffuse midline gliomas, will be critical in determining how aptamers might improve patient outcomes. In summary, aptamers represent a highly promising addition to the neuro-oncology toolkit—one that could complement and enhance existing diagnostic and therapeutic modalities. With continued interdisciplinary research and clinical validation, aptamers are poised to play a pivotal role in the future management of brain glial tumors, bringing us closer to more precise, effective, and individualized care for patients facing these challenging cancers.

## Acknowledgments

The work was supported by grants from the 10.13039/501100006769Russian Science Foundation project number 25-72-20052 to O.S.K. and the Ministry of Healthcare of the Russian Federation project 123031000006-4 to A.S.K.

## Author contributions

N.A.L. and T.N.Z. wrote the initial draft and created the figures. P.Z., G.S.Z., A.S.K., and A.A.N. contributed additional information and participated in manuscript editing. M.V.B. and O.S.K. edited the manuscript and supervised the work.

## Declaration of interests

The authors declare no competing interests.
